# Obsessional jealousy in a community sample: Association with relationship factors, impairment and perceived treatment needs

**DOI:** 10.1111/bjc.12409

**Published:** 2022-12-14

**Authors:** Johan Ahlen, Johan Bjureberg, Fabian Lenhard, Tove Wahlund, Johanna Linde, David Mataix‐Cols

**Affiliations:** ^1^ Department of Global Public Health Karolinska Institutet Stockholm Sweden; ^2^ Department of Clinical Neuroscience Karolinska Institutet Stockholm Sweden

**Keywords:** functional impairment, obsessional jealousy, perceived needs

## Abstract

**Objectives:**

Romantic jealousy could be understood as a continuum, from reality‐based, transient and functional jealousy to a more chronic form of jealousy with varying insight, intensity and duration. The latter has some overlaps with obsessive–compulsive disorder (here termed obsessional jealousy). Little is known about the nature of obsessional jealousy and its association with functional impairment, perceived negative consequences (drinking, violence), current and past relationship factors (e.g., length of relationship, being in love, infidelity, previous jealousy) or perceived need for professional support.

**Methods:**

Participants were 1076 adults (55% women) who filled in an anonymous survey.

**Results:**

Obsessional jealousy, measured with the Obsessional Jealousy Severity Scale, was strongly associated with functional impairment and verbal violence, and more weakly with physical violence and alcohol consumption. Individuals with a history of previous jealousy had more severe symptoms and were more likely to perceive the need for psychological support. Approximately 25% of the sample expressed interest in treatment.

**Conclusions:**

The results suggest that there is a group of individuals with impairing levels of obsessional jealousy who have a perceived need for help with their difficulties. More research is needed on the prevalence and clinical characteristics of these individuals. The development of jealousy‐specific psychological models and treatments is warranted.

**Practitioner points**

- There is a group of individuals with impairing levels of obsessional jealousy who have a perceived need for help with their difficulties.
- Few had previously been in contact with mental health professionals due to jealousy.
- The development of psychological models and treatments for obsessional jealousy is warranted.

## BACKGROUND

### Jealousy and negative consequences

Jealousy is a well‐recognized psychological state which includes negative feelings, suspicious or worrying thoughts about infidelity or loss of one's partner, and an urge to monitor or control one's partner or other romantically treasured persons (Chung & Harris, 2018; Martínez‐León et al., 2017). Previous research has predominantly focused on evolutionary and psychological aspects of jealousy, where researchers have, for example, explored potential gender differences in terms of jealousy over intimacy‐ and sexual infidelity (Sagarin et al., 2012). Cross‐sectional studies have explored the association between jealousy and a range of negative consequences. High levels of suspicious thoughts and worries about infidelity were found to be strongly associated with low relationship quality and satisfaction (Barelds & Barelds‐Dijkstra, 2007; Dandurand & Lafontaine, 2014). High levels of jealous behaviours (monitoring, controlling and surveillance) were found to be associated with cyber abuse (e.g., threatening, harassing, impersonating or humiliating using mobile phones, social media or e‐mails) and cyber stalking (Deans & Bhogal, 2019; Strawhun et al., 2013). Furthermore, Foran and O'Leary (2008) found that jealousy explained much of the variance in partner aggression. Finally, sexual jealousy has been found to be a common motive for homicides (Block & Block, 2012); according to a report from the Swedish Crime Prevention Council, jealousy was the second leading cause of men's lethal violence against women in close relationships (Rying, 2007).

### Relationship factors

A few studies have suggested that jealousy is positively associated with anxious and avoidant attachment (Guerrero, 1998; Rydell & Bringle, 2007). The direct association between jealousy and infidelity has not been studied. However, a positive association has been found between being the victim of infidelity and feelings of distress when being asked hypothetical infidelity questions (Burchell & Ward, 2011). A positive association between romantic love and jealousy was found in a small college sample (Mathes & Severa, 1981) and being in a relationship was negatively associated with distress to questions about hypothetical infidelity, in women only (Burchell & Ward, 2011).

### A clinical perspective

From a clinical perspective, research on what constitutes problematic jealousy (often referred to as morbid/pathological jealousy) has been fragmentary (De Silva, 1997; Tarrier et al., 1990). Morbid jealousy has mainly been described in the context of delusional jealousy sometimes occurring in psychoses (Batinic et al., 2013), though it seems likely that only a small proportion of individuals with morbid jealousy are psychotic (Easton et al., 2008). An obsessional type may be more common (Cobb & Marks, 1979; Tarrier et al., 1990). In fact, obsessional jealousy is mentioned in the DSM‐5 under ‘other specified obsessive‐compulsive and related disorders' (American Psychiatric Association, 2013).

Although clinicians encounter patients with obsessional jealousy (Kellett & Totterdell, 2013), and jealousy is a common reason for seeking couples therapy (De Silva, 1997), its prevalence is currently unknown (Kellett & Totterdell, 2013; Kingham & Gordon, 2004). There is no established evidence‐based treatment for obsessional jealousy. To our knowledge, only a handful case studies and uncontrolled trials exploring cognitive behavioural therapy and cognitive analytic therapy have been conducted (Cobb & Marks, 1979; Curling et al., 2018; Kellett & Totterdell, 2013). A search on Google Scholar using the phrase ‘treatment for jealousy’ conducted on the 23rd of June 2022 resulted in only ten hits. It is unclear whether individuals who have obsessional jealousy want help and if so, which kind of help.

In the current cross‐sectional study, we used an open survey to gather preliminary data on the nature of obsessional jealousy in the community and its association with functional impairment, perceived negative consequences (problematic drinking habits and violence), past and current relationship factors (e.g., length of relationship, being in love and infidelity) and perceived need for help. We hypothesized that obsessional jealousy would be significantly positively associated with functional impairment, problematic drinking and violence. Furthermore, we hypothesized that obsessional jealousy would be negatively associated with length of relationship, and positively associated with being in love, current and previous experiences of infidelity and jealousy in previous relationships. We also predicted that obsessional jealousy would be associated with higher perceived need for help.

## METHODS

### Participants

A total of 2321 participants opened the online survey by clicking on a link on the Karolinska Institute's homepage. A total of 1087 of these (49%) completed the survey. No information was collected from those who did not complete the survey. The survey was set to only allow one response from the same computer. To exclude random careless responses, we used the semantic synonym technique identifying respondents with inconsistent responses to similar items. We excluded 11 participants that gave inconsistent responses on their relationship status (e.g., choosing the response ‘in a steady relationship’ and ‘not in a current relationship’ on two different items). Consequently, the final study sample comprised 1076 participants, ranging between 18 and 80 years in age (*M* = 43.4, *SD* = 12.6). The gender distribution was roughly equal (55% women), and most were born in Sweden (93%). See Table 1 for further characteristics of the study sample.

**TABLE 1:** Study sample characteristics

Variable	Categories	*N* (%)
Gender	Women	595 (55%)
	Men	454 (42%)
	Other/uncertain/do not want to answer	27 (3%)
Age	18–29 years	188 (17%)
	30–39 years	226 (21%)
	40–49 years	292 (27%)
	50–59 years	251 (23%)
	60+ years	103 (10%)
Birth Country	Sweden	1003 (93%)
	Abroad	73 (7%)
Educational attainment	≤9 years	36 (3%)
	10–12 years	307 (29%)
	>12 years	733 (68%)
Employment	Employed/self‐employed	768 (71%)
	Student	117 (11%)
	Retired	74 (7%)
	Sick leave/parental leave	54 (5%)
	Jobseeker	45 (4%)
	Other	18 (2%)
Relationship status	Steady	739 (69%)
	Dating	87 (8%)
	Complicated/undefined	16 (1%)
	Poly‐relationship	38 (4%)
	Single	196 (18%)
Sexual orientation	Heterosexual	849 (79%)
	Bisexual	121 (11%)
	Pansexual	36 (3%)
	Gay/lesbian	33 (3%)
	Other /do not want to answer	37 (3%)
Previous contact for mental health issues	Yes	737 (68%)
	No	339 (32%)

### Procedure

We recruited participants using a (in Swedish language) Facebook advertisement including the following information ‘Jealousy is something that most of us have experienced at some point in life. You are welcome to participate in our survey, regardless of whether you are jealous or not. Your answers will be completely anonymous’. Participants, who clicked on the advertisement, were linked to a homepage at Karolinska Institutet where they could read about the study and access the web survey on surveymonkey.com. To start the survey, participants were required to read information about the study, confirm being 18 years or above and consent to participating in the study. The web survey was open for about one month between September 10th and October 4th in 2020. Initially, a vast majority of the participants who answered the survey were highly educated women. To reach a more representative sample, we eventually adjusted the advertisement to only reach people in the eight regions (out of 21) in Sweden with the (on average) lowest level of education. During the last two weeks of data collection, the ad was additionally adjusted to only reach men, to increase the proportion of men in the study sample. The Swedish Ethical Review Authority had no ethical concerns about this study (Dnr 2020‐00985).

### Measures

#### The Obsessional jealousy Severity Scale

The recently developed Obsessional jealousy Severity Scale (OJSS; Ahlen et al., 2022) was modelled on the Yale‐Brown Obsessive Compulsive Scale (Goodman et al., 1989) and, unlike other instruments, is specifically designed to capture the clinical aspects of jealousy (i.e., whether jealousy causes distress and disrupts functioning). The OJSS is divided into two sections, (1) jealous thoughts and (2) jealous behaviours; both sections include a checklist and a severity scale. Respondents are informed that they may have jealous thoughts/behaviours about someone that they have, have had or wish to have a romantic relationship with (marked as ‘X’ in the checklist examples). In the jealous 17‐item thoughts checklist, respondents report if they recognize (yes/no) a series of jealous thoughts, for example ‘I suspect or worry that X is attracted to someone else’. In the severity scale for jealous thoughts, respondents are asked to indicate on a Likert scale from 0 to 4 how frequent, impairing, and distressing these jealous thoughts have been during the past week, whether he/she has tried to resist these thoughts, and what control he/she has over them. The respondents follow the same procedure regarding jealous behaviours, that is first respond to a checklist of 32 jealous behaviours (for example ‘I check X's phone’, or ‘I forbid X to go the pub without me’), and then according to the severity scale indicate frequency, impairment, distress, effort to resist and control of these jealous behaviours during the past week. The total scores of the two severity scales are summed to a total jealousy severity score. Scores range from 0 to 40, with higher scores denoting greater severity. A preliminary psychometric evaluation of a self‐rated version of the OJSS showed adequate convergent and divergent validity, measurement invariance, and an excellent internal consistency (Ahlen et al., 2022). In the current study sample, Cronbach's alpha of the 10 severity items was = .92.

#### The Work and Social Adjustment Scale (WSAS)

The WSAS is a self‐rated questionnaire measuring functional impairment that is frequently used in psychiatry (Mundt et al., 2002). The version used in the current study measured impairments specifically caused by jealousy. The brief questionnaire contains five questions rated between 0 (not at all impaired) and 8 (very severely impaired) and covers five life areas; work, home management, social leisure activities, private leisure activities and close relationships. The WSAS has shown convergence with disorder severity (obsessive–compulsive disorder [OCD] and depression) and good reliability (test–retest correlation; Mataix‐Cols et al., 2005; Mundt et al., 2002).

#### The Alcohol Use Disorders Identification Test (AUDIT)

AUDIT is a validated 10‐item instrument used for screening of harmful alcohol use and alcohol dependence (Saunders et al., 1993). The items cover alcohol consumption, drinking behaviours and alcohol‐related problems in the past year. Each item is scored from 0 to 4. In the current sample, Cronbach's alpha was = .84.

#### Violent behaviour

To assess any violent behaviour caused by jealousy, we included three questions covering verbal, physical and sexual violence. Respondents were asked if jealousy (of the respondent) leads to quarrel or heated discussions, physical violence or sexual violence, rated on a Likert scale ranging from 0 to 3 (0 = Never, 3 = Almost always).

#### Relationship factors

We included questions exploring the participants' current and previous relationships. Four questions explored the current relationship: relationship status (single, dating, steady relationship, poly‐relationships or complicated/undefined relationships), length of current relationship (>3 months, 3–12 months, 1–5 years, 5–10 years, > 10 years or no current relationship), how in love they were (not at all, a bit, pretty much, much or no current relationship) and experience of infidelity in the current relationship (participant unfaithful, partner unfaithful, both participant and partner unfaithful, no infidelity or no current relationship). Two further questions explored previous relationships: infidelity in previous relationships (participant unfaithful, partner unfaithful, both participant and partner unfaithful, no infidelity in previous relationships or no previous relationships) and whether they had been jealous in previous relationships (never, seldom, sometimes, often, always or no previous relationships).

### Data analysis

All analyses were performed in the *R* software (R Core Team, 2017). To examine differences in jealousy severity between groups we performed one‐way ANOVAs. In models with statistically significant differences, we applied post‐hoc analyses (Tukey‐HSD) to further explore these differences. The magnitude of differences was described in terms of effect sizes (i.e., Cohen's *d*) calculated by dividing the difference in jealousy severity by the standard deviation of the total sample. According to guidelines, Cohen's *d* ≥ .2 is typically interpreted as small effect, Cohen's *d* ≥ .5 moderate effect and Cohen's *d* ≥ .8 a large effect (Cohen, 1977).

Associations between jealousy severity (as measured by OJSS), impairment and negative consequences were analysed using Spearman rank‐order correlation. The strength of associations was also interpreted using Cohen's guidelines where a correlation coefficient (*r*) ≥ .1 is typically interpreted as a small association, an *r* ≥ .3 a moderate association and a *r* ≥ .5 a strong association (Cohen, 1977).

## RESULTS

### Jealousy and personal characteristics

In Table 2, we present mean values of jealousy severity, as measured by the OJSS, stratified by personal characteristics. In a series of ANOVAs, we found that age, educational attainment, relationship status, sexual orientation and previous mental health care all were associated with jealousy severity scores. Specifically, young adults, individuals with low educational attainment, single, identified as gay or lesbian and those with a history of previous contact with mental health services had higher jealousy severity scores. Conversely, country of birth, sex and employment status were not significantly associated with jealousy severity.

**TABLE 2:** Mean and standard deviations of the obsessional jealousy severity scale, divided by personal characteristics

Variable	OJSS	*p*‐Value^a^
	*M* (*SD*)	
Gender		
Women	6.3 (6.0)	.27
Men	5.7 (5.6)	
Other/uncertain/do not want to answer	5.4 (6.2)	
Age		
18–29 years	8.2 (6.7)	<.001***
30–39 years	5.6 (5.7)	
40–49 years	6.0 (5.4)	
50–59 years	5.6 (5.7)	
60+ years	4.0 (4.8)	
Birth Country		
Sweden	5.9 (5.7)	.05
Abroad	7.3 (7.0)	
Educational attainment		
≤9 years	7.8 (6.5)	<.001***
10–12 years	6.7 (6.1)	
>12 years	5.6 (5.6)	
Employment status		
Employed/self‐employed	5.9 (5.7)	.67
Student	6.6 (6.1)	
Retired	5.8 (6.2)	
Sick leave/parental leave	5.9 (6.1)	
Jobseeker	6.9 (5.6)	
Other	6.8 (6.2)	
Relationship status		
Steady	5.6 (5.7)	<.001***
Dating	7.0 (6.3)	
Complicated/undefined	8.8 (5.6)	
Poly‐relationship	4.4 (4.8)	
Single	7.1 (5.9)	
Sexual orientation		
Heterosexual	6.2 (6.0)	.002**
Bisexual	5.1 (4.9)	
Pansexual	3.8 (4.1)	
Gay/Lesbian	8.6 (5.9)	
Other/do not want to answer	5.1 (5.6)	
Any previous contact for mental health issues		
Yes	6.3 (6.0)	.01*
No	5.3 (5.5)	

Abbreviation: OJSS, Obsessional Jealousy Severity Scale.

*p < .05, **p < .01, ***p < .001.

^a^ *p*‐Value from ANOVA.

### Jealousy, impairment and negative consequences

A series of Spearman rank‐order correlation analyses showed statistically significant positive correlations between jealousy severity and impairment in all life areas explored (see Table 3). All correlations were interpreted as strong.

**TABLE 3:** Spearman rank correlation between OJSS, impairments in different life areas, violent behaviour and alcohol consumption

Associated variable	OJSS
WSAS – Work	.60***
WSAS – Home management	.53***
WSAS – Social leisure	.63***
WSAS – Private leisure	.64***
WSAS – Close relationships	.63***
Verbal violence	.51***
Physical violence	.17***
Sexual violence	.03
AUDIT	.13***

*Note*: ***p < .001.

Abbreviations: AUDIT, The Alcohol Use Disorders Identification Test; OJSS, Obsessional Jealousy Severity Scale; WSAS, Work and social adjustment scale.

Jealousy severity was also strongly associated with verbal violence, but only weakly to physical violence and alcohol consumption. We found no association between jealousy severity and sexual violence (Table 3).

### Jealousy and relationship factors

Table 4 presents the distribution of participants according to relationship length, being in love, infidelity in their current relationship, infidelity in previous relationships and jealousy in previous relationships.

**TABLE 4:** Distribution of participants according to relationships factors

Variable	Categories	*N* (%)
Relationship length	<3 months	55 (5%)
	3–12 months	111 (10%)
	1–5 years	257 (24%)
	5–10 years	154 (14%)
	>10 years	317 (29%)
	Not relevant	182 (17%)
Being in love with partner	Not at all	38 (4%)
	A bit	131 (12%)
	Pretty much	235 (22%)
	Much	496 (46%)
	No current relationship	176 (16%)
Unfaithful in current relationship	Me	108 (10%)
	Partner	95 (9%)
	Me and partner	36 (3%)
	Neither me, nor partner	636 (59%)
	No current partner	201 (18%)
Unfaithful in previous relationships	Me, but not partners	145 (13%)
	Previous partner(s) but not me	278 (26%)
	Both me and previous partner(s)	363 (34%)
	Not me, not previous partner(s)	290 (27%)
Jealous in previous relationships	Never	160 (15%)
	Seldom	418 (39%)
	Sometimes	314 (29%)
	Often	119 (11%)
	Always	44 (4%)
	No previous relationships	21 (2%)

#### Relationship length

An ANOVA showed significant differences in jealousy severity between groups with different relationship length, *F*(5, 1070) = 7.51, *p* < .001. A post hoc analysis showed that individuals in relationships longer than 10 years had lower levels of jealousy compared to those without a current relationship (*d* = .35, *p* = .002), a relationship that had lasted between 3–12 months (*d* = .48, *p* < .001) and 1–5 years (*d* = .40, *p* < .001).

#### Being in love

An ANOVA showed no significant differences in jealousy severity between groups with different levels of being in love, *F*(4, 1071) = .41, *p* = .80.

#### Infidelity in current relationship

An ANOVA showed significant differences in jealousy severity between groups with experiences of infidelity in their current relationship, *F*(4, 1071) = 10.94, *p* < .001. A post hoc analysis showed that individuals who answered that neither them nor their partner had been unfaithful in the current relationship had moderately lower levels of jealousy compared to those whose current partner (*d* = .51, *p* < .001), themselves (*d* = .41, *p* = .002) or both had been unfaithful (*d* = .50, *p* = .03), and also somewhat lower levels of jealousy compared to those with no current relationship (*d* = .30, *p* = .002).

#### Previous experiences of infidelity

An ANOVA showed significant differences in jealousy severity between groups with experiences of infidelity in previous relationships, *F*(3, 1072) = 4.17, *p* = .006. A post‐hoc analysis showed that individuals with experiences of an unfaithful partner in previous relationships had slightly higher levels of jealousy compared to those with no experiences of infidelity in previous relationships (*d* = .27, *p* = .02) and to those who themselves had been unfaithful (*d* = .25, *p* = .04).

#### Previous jealousy

**FIGURE 1:**
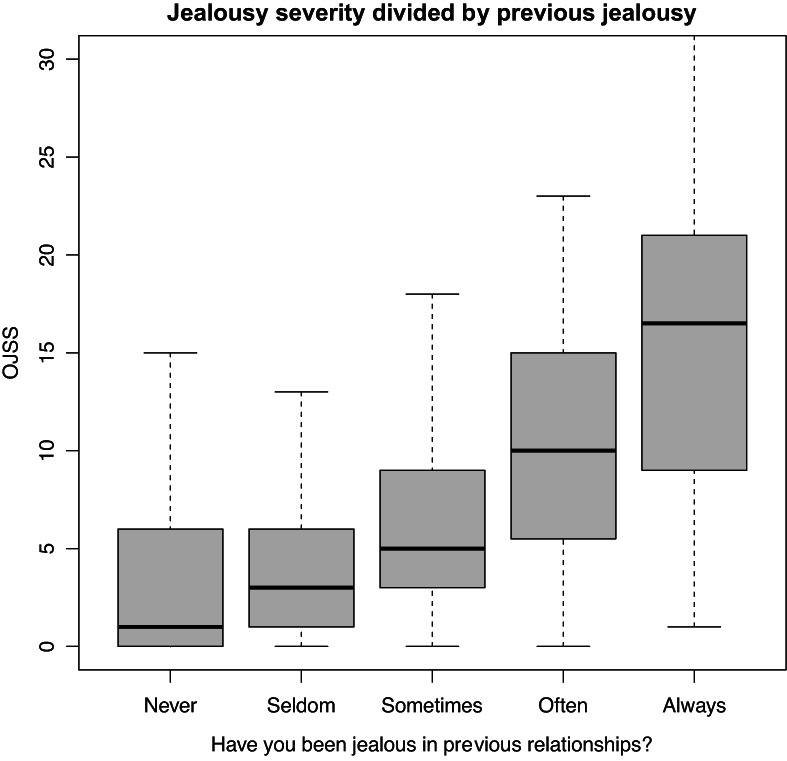
Jealousy severity divided by jealousy in previous relationship

An ANOVA showed significant differences in jealousy severity between groups with different levels of jealousy in previous relationships, *F*(5, 1070) = 62.92, *p* < .001. A post‐hoc analysis showed moderate to very large differences in jealousy severity between all levels of previous jealousy (except for those who never or seldom had been jealous in previous relationships). For example, those who reported that they always had been jealous in previous relationships had higher jealousy compared to those who often (*d* = .89, *p* < .001), sometimes (*d* = 1.52, *p* < .001), seldom (*d* = 1.90, *p* < .001) or never (*d* = 2.04, *p* < .001) had been jealous. OJSS values for different groups of previous jealousy are visualized in Figure 1.

### Jealousy and need for help

**FIGURE 2:**
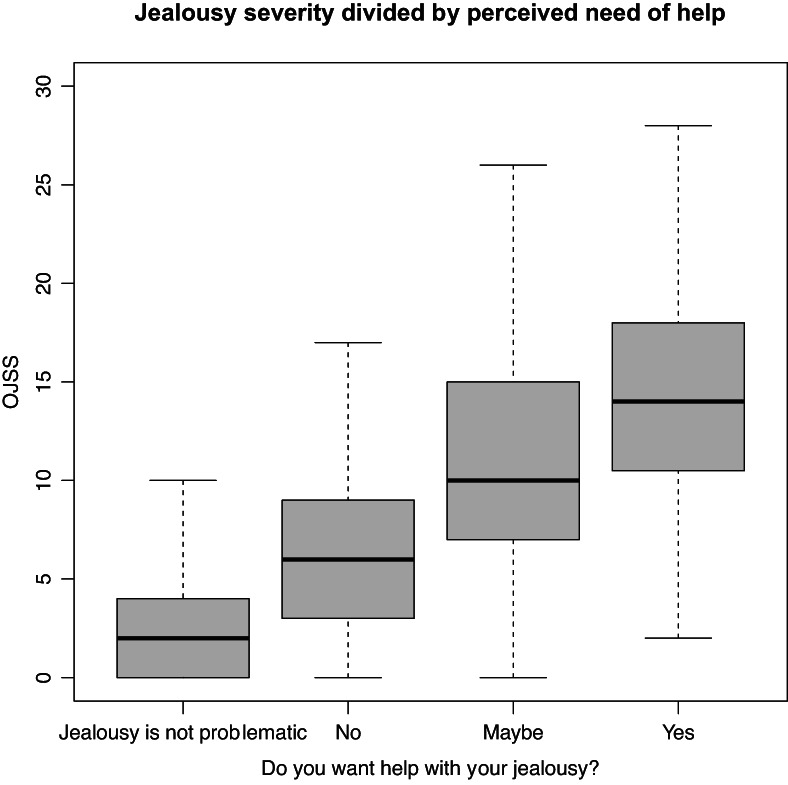
Jealousy severity divided by perceived needs of help

Of the total sample, 88 (8%) reported that they wanted, and 183 (17%) that they maybe wanted help with their jealousy. The remaining participants reported that they did not want help (*n* = 226, 21%) or that their jealousy was not problematic (*n* = 579, 54%). OJSS values for different groups of perceived need for help are visualized in Figure 2.

Of the 271 participants who wanted or maybe wanted help, 37 (14%) had experienced partner infidelity in their current relationship. Similarly, of the 805 participants who did not want help (or reported that jealousy was not a problem), 94 (12%) had experienced partner infidelity in their current relationship. A chi‐square test showed no statistically significant difference between these proportions, *X* ^2^(1, 1076) = .58, *p* = .45.

Of the 271 participants who wanted or maybe wanted help, only 44 (16%) had previously sought help for jealousy, while 204 (75%) had previously sought professional help for other mental health issues. On an open‐ended question, 163 of those who wanted or maybe wanted help noted reasons for previous contact with mental health care providers. We categorized reasons for previous contact with mental health care providers into four groups; relationship problems; anxiety, depression and stress‐related disorders; obsessive–compulsive and related disorders; and other psychiatric disorders (such as personality disorder, bipolar disorder, autism spectrum disorder, ADHD, substance use disorders or psychosis). The majority (*n* = 120) reported reasons related to anxiety, depression and stress‐related disorders, while 27 reported other psychiatric conditions. Eleven reported relationship problems, and five reported OCD and related disorders.

Of the 88 participants who wanted help with their jealousy, 83 would consider psychological treatment if it was available, and the remaining five would maybe consider it.

## DISCUSSION

In this open survey, we explored obsessional jealousy in the community and its association with functional impairment, perceived negative consequences (violent behaviour and alcohol), relationship factors and perceived need for help. We found a strong association between obsessional jealousy and functional impairment in different life areas, verbal violence, and relationship factors and about a quarter of participants who wanted or maybe wanted help with their jealousy.

The strong association between jealousy and verbal violence and the small association between jealousy and physical violence are in line with previous research (Kar & O'Leary, 2013).

With regard to relationship factors, the strongest association found was between (current) jealousy and being jealous in previous relationships. This suggests that clinical levels of jealousy may constitute a durable trait. However, infidelity in the current relationship was moderately associated to jealousy, which suggest that jealousy may also be affected by the current relationship situation. Notably, other relationship factors, that is infidelity in previous relationships, length of the relationship, and how strongly in love one was, were only marginally associated with jealousy severity.

Approximately 25% of the sample stated that they would definitely or might need help for their jealousy. A somewhat unexpected finding was that only a small proportion of these had experiences of partner infidelity in their current relationship. Thus, the perceived need of help for jealousy was not driven by infidelity in the current relationship. Few of the participants with a perceived need of help had previously been in contact with professionals due to jealousy, but many had sought help for other mental health issues, mainly anxiety and depression. Very few reported previous contacts with mental health facilities due to psychotic‐ or alcohol‐related issues, which is in line with previous work (Tarrier et al., 1990). Given the current study design, we cannot comment on the prevalence of individuals with a perceived need of help in the general population. However, the fact that we could identify a substantial proportion of participants who wanted or maybe wanted help in only one month of data collection and with a small budget for advertisements suggests that the prevalence may not be negligible. More systematic study of the prevalence of obsessional jealousy in the general population using probabilistic sampling methods would be an important next step.

The results of this study suggest that the development of psychological interventions for obsessional jealousy may be a worthwhile endeavour. In future treatment development, experiences from previous case studies, mainly based on a cognitive–behavioural framework, are probably valuable (Cobb & Marks, 1979; De Silva, 1997). The suffering and functional impairment caused by jealous thoughts and behaviours bears similarities to OCD, and in many cases seems to follow a chronic course, speaking for exposure and response prevention as potentially effective treatment components for jealousy (Ecker, 2012). Treatment may also include fostering relationships skills such as identifying negative patterns when interacting with the partner, handling conflicts in a constructive way (explicitly addressing verbal and physical violence), increasing acceptance of each other's differences and creating more closeness in the relationship. In the assessment and treatment of individuals who express jealousy, aggressive tendencies should be investigated and targeted. In addition, jealousy might be relevant to assess and address in aggressive individuals.

### Limitations

The current study had an exploratory approach, using an open survey, which is associated to several threats to the validity of the conclusions. First, despite our efforts to recruit a representative sample of the Swedish population, the non‐probabilistic sampling method means that the sample may not have been representative. Our sample had a higher educational level compared with the general Swedish population, and two out of three participants reported that they at some point had sought professional help for mental health issues, which is higher than expected in the general population in Sweden (Forslund et al., 2020). Further, due to the self‐reported nature of the study, we did not include questions on whether the respondents believed their jealous thoughts were justified or rational, which limited us from exploring the level of conviction or insight in jealous individuals. Although the anonymous self‐reported approach might have had a positive effect on the validity of the data (given the stigmatizing nature of jealousy), future studies would benefit from recruiting treatment‐seeking samples and using clinician‐rated measures of insight.

## CONCLUSION

With these limitations in mind, the current study suggests that there is a group of individuals with impairing levels of obsessional jealousy who have a perceived need for help with their difficulties. More research is needed on the prevalence and clinical characteristics of ‘obsessional jealousy disorder’, should a new diagnostic category emerge in the future as being clinically useful. Regardless of nosological issues, the development of jealousy‐specific psychological models and treatments is warranted.

## AUTHOR CONTRIBUTIONS

**Johan Ahlen:** Conceptualization; formal analysis; investigation; methodology; project administration; writing – original draft. **Johan Bjureberg:** Conceptualization; methodology; writing – review and editing. **Fabian Lenhard:** Conceptualization; writing – review and editing. **Tove Wahlund:** Conceptualization; writing – review and editing. **Johanna Linde:** Writing – review and editing. **David Mataix‐Cols:** Conceptualization; methodology; supervision; writing – review and editing.

## CONFLICTS OF INTEREST

We have no conflicts of interest to disclose.

## Data Availability

Data can be shared upon reasonable request from collaborators. Analysis code for the study is available by emailing the corresponding author.
